# Platelet-Rich Plasma Versus Bone Marrow Aspirate Concentrate for Hip Disorders: A Narrative Review of Biological Rationale, Clinical Evidence, and Research Priorities

**DOI:** 10.3390/bioengineering13091018

**Published:** 2026-09-01

**Authors:** Se Yeong Jeon, Chul Hee Jung, Seok-Yeon Choi, Dong Ha Lee

**Affiliations:** 1 Science and Technology Department, Ulsan National Institute of Science and Technology, Ulsan 44919, Republic of Korea; skiende74@gmail.com; 2 Department of Orthopaedic Surgery, Aerospace Medical Center, Republic of Korea Air Force, Cheongju 28187, Republic of Korea; chulh2006@gmail.com; 3 Department of Rehabilitation Medicine, Aerospace Medical Center, Republic of Korea Air Force, Cheongju 28187, Republic of Korea; csyceo@naver.com; 4 Department of Orthopaedic Surgery, Seoul National University Hospital, 101 Daehak-ro, Jongno-gu, Seoul 03080, Republic of Korea

**Keywords:** hip osteoarthritis, osteonecrosis of the femoral head, gluteal tendinopathy, platelet-rich plasma, bone marrow aspirate concentrate, orthobiologics, narrative review

## Abstract

Hip disorders—including hip osteoarthritis (OA), greater trochanteric pain syndrome (GTPS) with gluteal tendinopathy, femoroacetabular impingement syndrome (FAIS) with associated labral pathology, and osteonecrosis of the femoral head (ONFH)—are common causes of pain and functional loss, and several are refractory to conventional non-operative care. Platelet-rich plasma (PRP) and bone marrow aspirate concentrate (BMAC) are the two most widely used autologous orthobiologics, yet a comparative appraisal of these agents across the full spectrum of hip pathology is lacking. This narrative review is based on structured searches of MEDLINE, Embase, Cochrane Central Register of Controlled Trials (CENTRAL), and Scopus for clinical studies—randomized controlled trials (RCTs), comparative studies, and prospective series—and evidence syntheses evaluating PRP or BMAC in hip disorders; studies were synthesized narratively by biologic type and by disorder, and no formal systematic-review or scoping-review reporting protocol was applied. Society guidelines and consensus documents were additionally examined, and PRP formulation subgroups (leukocyte-rich [LR-PRP] versus leukocyte-poor [LP-PRP]) were considered, including a numerically larger, but methodologically heterogeneous and comparator-sensitive evidence base for hip disorders, supported by several RCTs and pooled analyses in hip OA and in gluteal tendinopathy; however, findings are heterogeneous, effect sizes are modest, a network meta-analysis found intra-articular saline to be statistically indistinguishable from PRP for hip OA pain, and the only adequately powered placebo-controlled RCT in GTPS was negative. BMAC evidence in the hip is concentrated almost entirely in surgical augmentation of core decompression for ONFH, where results are stage-dependent and include a negative double-blind RCT in post-collapse disease; intra-articular BMAC for hip OA rests on small uncontrolled cohorts, and no standalone hip BMAC RCT has been published. No RCT directly compares PRP with BMAC for any hip disorder; the closest evidence is a knee OA meta-analysis, in which both biologics outperformed hyaluronic acid with no clear separation between the two. Current society guidance advises strongly against PRP for hip OA, and no guideline currently incorporates BMAC for hip indications. Neither biologic is established as superior. The search for standardized, hip-specific, adequately powered head-to-head RCTs incorporating biologic characterization, structural (imaging) endpoints, and longer follow-up, represents the most important research priority in this field.

## 1. Introduction

Osteoarthritis (OA) is the most common form of arthritis in adults and a leading contributor to years living with a disability worldwide. Global Burden of Disease modelling estimates that, relative to 2020, hip OA cases will rise by approximately 79% by 2050, with high body mass index (BMI) accounting for roughly one-fifth of the attributable burden [1]. The hip is the second most frequently affected weight-bearing joint after the knee, and hip disease encompasses more than intra-articular cartilage loss: greater trochanteric pain syndrome (GTPS) arising from gluteus medius and minimus tendinopathy, femoroacetabular impingement syndrome (FAIS) with acetabular labral pathology, and osteonecrosis of the femoral head (ONFH) each contribute substantially to hip-related morbidity, often in younger and more active patients.

Hip osteoarthritis develops through the convergence of mechanical, developmental, and cellular-inflammatory mechanisms. The dominant mechanical risk factors for primary hip OA are obesity, advanced age, and developmental morphological variants—including femoroacetabular cam and pincer deformity and developmental dysplasia of the hip (DDH)—each of which alters load distribution across the articular surface. At the cellular level, chondrocyte dysfunction driven by dysregulation of the NF-κB and TGF-β signalling pathways results in upregulation of matrix metalloproteinases (MMPs) and a disintegrin and metalloproteinase with thrombospondin motifs (ADAMTS-5), leading to progressive proteoglycan loss and type II collagen degradation. Synovial macrophage polarisation toward the pro-inflammatory M1 phenotype amplifies this catabolic environment through the secretion of interleukin-1β (IL-1β) and tumour necrosis factor-α (TNF-α) 1. This mechanistic framework is directly relevant to the therapeutic rationale for PRP and BMAC, both of which carry anti-inflammatory and trophic mediators that may—at least in theory—modulate these pathological cascades.

Conventional non-operative management—education, structured exercise, weight optimization, oral and topical non-steroidal anti-inflammatory drugs (NSAIDs), and intra-articular corticosteroid (CS) injection—relieves symptoms but does not modify the underlying pathology. Society guidance reflects this: the 2019 American College of Rheumatology (ACR)/Arthritis Foundation guideline and the 2019 Osteoarthritis Research Society International (OARSI) recommendations centre on core non-pharmacological treatment with a narrow role for injectable agents in the hip [2,3]. The therapeutic gap between failed conservative care and total hip arthroplasty (THA) is wide, particularly for patients who are young, who have early radiographic disease, or who wish to defer arthroplasty. This gap has driven rapid, largely market-led adoption of autologous orthobiologics.

Two products dominate clinical practice. Platelet-rich plasma (PRP) is an autologous plasma fraction enriched in platelets and their α-granule growth factors—including platelet-derived growth factor (PDGF), transforming growth factor-β (TGF-β), and vascular endothelial growth factor (VEGF)—prepared at the point of care from peripheral blood; upon activation, these mediators are released to modulate inflammation, cell proliferation, and matrix synthesis [3]. Bone marrow aspirate concentrate (BMAC) is a centrifuged fraction of autologous bone marrow aspirate (BMA) containing nucleated cells, hematopoietic and mesenchymal stromal cell (MSC) progenitors, platelets, and a broad soluble milieu. The two are frequently discussed as interchangeable “regenerative injections,” yet they differ fundamentally in cellular content, harvest morbidity, cost, regulatory status, and—critically—in the quantity and quality of hip-specific clinical evidence supporting them.

For PRP, the hip literature includes multiple RCTs and pooled analyses in hip OA, in FAIS surgery, and in gluteal tendinopathy, although a Bayesian network meta-analysis reported that intra-articular saline performed comparably to CS, hyaluronic acid (HA), and PRP for hip OA pain at up to six months [4], and preparation protocols across trials are heterogeneous and inconsistently reported [5]. For BMAC, the hip evidence base is much thinner and is skewed toward surgical augmentation of core decompression (CD) for ONFH, a technique first popularized by Hernigou and Beaujean, rather than toward standalone intra-articular injection [6,7]. No published RCT directly compares PRP with BMAC for any hip disorder; the nearest randomized evidence comes from the knee, where both biologics outperformed HA in a Level I meta-analysis without a decisive separation between them [8].

The purpose of this narrative review is therefore threefold: (i) to compare the biological rationale, composition, and preparation variability of PRP and BMAC as applied to the hip; (ii) to appraise the hip-specific clinical evidence for each agent, disorder by disorder, including PRP formulation subgroups; and (iii) to define the methodological and regulatory priorities that must be addressed before either biologic can be positioned in an evidence-based hip treatment algorithm. Prior reviews have addressed PRP or BMAC individually for single hip disorders—hip OA [9,10,11,12,13,14,15,16,17] or ONFH cell therapy [18]—or have compared orthobiologics in the knee [8,9]; no published review has undertaken a disorder-by-disorder, comparative appraisal of both PRP and BMAC across the full spectrum of hip pathology within a single work. A further novel contribution is the explicit recognition that the two agents inhabit almost entirely separate clinical literatures—PRP predominantly as an injectable for soft-tissue and articular indications, BMAC predominantly as a surgical bone augment in ONFH—and that treating them as interchangeable “regenerative injections” conflates agents with fundamentally different biological modes of action.

## 2. Materials and Methods

This work is a narrative review. Structured searches of MEDLINE, Embase, the Cochrane Central Register of Controlled Trials (CENTRAL), and Scopus were performed for clinical studies (RCTs, comparative studies, and prospective series) and evidence syntheses evaluating PRP or BMAC in hip disorders. Search concepts combined terms for the anatomical site and pathology (hip, coxarthrosis, hip osteoarthritis, femoroacetabular impingement, acetabular labrum, greater trochanteric pain syndrome, gluteal tendinopathy, osteonecrosis/avascular necrosis of the femoral head) with terms for the interventions (platelet-rich plasma, PRP, autologous conditioned plasma, bone marrow aspirate concentrate, BMAC, bone marrow concentrate, bone marrow mononuclear cells). Reference lists of retrieved syntheses were hand-searched. English-language human clinical studies were prioritized; laboratory and animal work was cited only where it was required to explain the mechanism. No formal date restriction was imposed; however, the search was primarily directed at studies published from 2000 onwards, reflecting the modern clinical development of both products, and was updated to include literature available through July 2026. Study selection was performed independently by two investigators, with disagreements resolved by discussion.

Studies were synthesized narratively by biologic type and by disorder. No formal systematic-review or scoping-review reporting protocol (for example, the Preferred Reporting Items for Systematic Reviews and Meta-Analyses [PRISMA] statement or its scoping-review extension) was applied, no protocol was registered, no duplicate screening or formal risk-of-bias assessment was performed, and no quantitative pooling was undertaken. Current society guidelines and consensus statements were additionally examined, in order to position each biologic relative to standard care. Where trials characterized their PRP product, formulation subgroups—leukocyte-rich PRP (LR-PRP) versus leukocyte-poor PRP (LP-PRP)—were recorded and considered separately. The limitations inherent in this design are stated explicitly in Section 8.

## 3. Biological Rationale and Product Characterization

### 3.1. Platelet-Rich Plasma

PRP is produced by centrifugation of anticoagulated autologous whole blood to yield a plasma fraction with a supraphysiological platelet concentration. Upon activation, platelet α-granules release a coordinated set of mediators including platelet-derived growth factor (PDGF), transforming growth factor-β (TGF-β), vascular endothelial growth factor (VEGF), insulin-like growth factor-1 (IGF-1), epidermal growth factor (EGF), and basic fibroblast growth factor (bFGF). PDGF drives chemotaxis and cell proliferation; TGF-β modulates extracellular matrix synthesis and exerts anti-inflammatory effects by suppressing pro-inflammatory cytokine release; VEGF promotes angiogenesis in ischaemic or poorly vascularised tissue; and IGF-1 stimulates chondrocyte anabolism and inhibits apoptosis [10,11,12,13,14,15,16,17,18]. Taken together, these mediators create a local microenvironment that is plausibly favourable to tissue healing across multiple musculoskeletal compartments. These regulate chemotaxis, angiogenesis, cell proliferation, and matrix synthesis, and, in the arthritic joint, may additionally modulate synovial inflammation and chondrocyte anabolism [10].

Importantly, “PRP” denotes a family of products, rather than a single agent. Preparations differ in platelet dose, leukocyte content, erythrocyte contamination, activation state, injected volume, and number of treatments. These include the following: PAW (Platelets, Activation, White cells) [11], PLRA (Platelet concentration, Leukocyte content, Red blood cell content, Activation) [12], DEPA (Dose of injected platelets, Efficiency of production, Purity, Activation) [13], and MARSPILL (Method, Activation, Red blood cells, Spin, Platelets, Image guidance, Leukocytes, Light activation) [14]. More recently the Scientific and Standardization Committee of the International Society on Thrombosis and Haemostasis (ISTH) proposed a consolidated four-category scheme based on leukocyte and erythrocyte content [15]. From a standardisation and evidence-generation standpoint this variability makes trials difficult to compare. From a clinical and precision-medicine standpoint, however, this tunability is equally a feature: the capacity to tailor platelet dose, leukocyte content, and activation state to the individual patient’s target tissue and inflammatory phenotype aligns with the emerging paradigm of precision orthobiologics [15,16]. Active efforts to match preparation to indication—LP-PRP for intra-articular use, LR-PRP for tendinopathic tissue—represent exactly this personalized approach [16].

Two empirical findings anchor the clinical significance of this heterogeneity. First, a systematic review of the orthopaedic literature found that composition reporting was so incomplete that most published PRP studies could not be replicated or meaningfully compared [16]. Second, a head-to-head laboratory comparison of four widely used commercial kits demonstrated substantial differences in the platelet, leukocyte, and erythrocyte content of the final product from the same donor blood [17]. Any comparison of PRP with BMAC—or of PRP trials with one another—must be read with this in mind.

### 3.2. Bone Marrow Aspirate Concentrate

BMAC is obtained by percutaneous aspiration of bone marrow, most commonly from the posterior superior iliac spine, followed by density-gradient or buoyancy-based centrifugation to concentrate the nucleated cell fraction. The resulting product contains hematopoietic stem and progenitor cells, endothelial progenitors, monocytes, platelets, and a small population of connective tissue progenitors, of which culture-expanded derivatives satisfy the International Society for Cellular Therapy (ISCT) minimal criteria for MSCs—plastic adherence, expression of CD105, CD73, and CD90 with absence of CD45, CD34, CD14/CD11b, CD79α/CD19, and human leukocyte antigen-DR (HLA-DR), and trilineage differentiation capacity [18]. It is important to be precise here: BMAC is not a purified MSC product. MSCs typically represent a very small fraction of the nucleated cells delivered, and the dominant cell populations are hematopoietic [19]. The proposed mechanism is therefore better framed as delivery of an intact marrow niche with paracrine, immunomodulatory, and trophic activity, rather than as structural cell replacement.

BMAC composition is even more variable than that of PRP, and much of the variance is operator-dependent. Aspiration volume is critical: drawing large volumes from a single site dilutes the aspirate with peripheral blood and reduces progenitor yield per millilitre [20]. Harvest site matters, with the posterior iliac crest outperforming the anterior crest for MSC yield [21]. Donor age, sex, systemic disease, and the specific concentration device add further variance. Systematic reviews of the clinical orthopaedic literature have repeatedly shown that this variance is not reported: fewer than one-third of studies provided quantitative measures of the delivered product, and no study reported protocol detail sufficient for replication [22,23].

### 3.3. Comparative Biology

PRP and BMAC overlap, in that both deliver platelet-derived growth factors, and BMAC contains a platelet fraction of its own. They differ in that BMAC additionally supplies nucleated cells, progenitor populations, and interleukin-1 receptor antagonist (IL-1Ra) at concentrations not achievable with PRP. This provides a plausible mechanistic argument for BMAC in conditions requiring osteogenic or osteochondral repair—ONFH being the paradigmatic example—while offering less obvious incremental advantage in predominantly inflammatory or tendinopathic conditions where PRP alone may suffice. Whether this theoretical distinction translates into clinical superiority in the hip is precisely the question the current evidence base cannot answer. A critical further complication is that comparing PRP with BMAC in the hip conflates agents that are being studied in fundamentally different clinical contexts: PRP primarily as an intra-articular or peritendinous injection for OA and tendinopathy, and BMAC primarily as an intralesional surgical augment for ONFH. These do not address the same biological question, and the products are not being asked to perform the same biological work. This contextual asymmetry is addressed further in Section 6. Table 1 summarizes the comparison.

## 4. Platelet-Rich Plasma in Hip Disorders

### 4.1. Hip Osteoarthritis

Across six randomized trials in hip OA, the consistent finding is that PRP is non-inferior to HA but has not been demonstrated to be superior to saline. Hip OA is the most studied PRP indication in the hip. Because the joint is deep, essentially all published trials used ultrasound guidance. Battaglia et al. compared ultrasound-guided PRP with HA in a randomized trial and reported comparable improvement between arms [24]. Dallari et al. randomized patients to PRP, HA, or PRP plus HA and found the PRP arm to perform favourably on pain outcomes, with the combination offering no clear additional benefit [25]. Di Sante et al. reported early pain reduction with PRP relative to HA that was not sustained at later follow-up [26]. Doria et al., in early hip OA, found PRP and HA to yield similar improvement [27]. Kraeutler et al. performed a double-blind randomized pilot comparing LP-PRP with low-molecular-weight HA and did not demonstrate superiority of either agent [28]. Okanoue et al. conducted a double-blind RCT in secondary hip OA arising from DDH, delivering three ultrasound-guided injections; PRP produced a modestly greater improvement in visual analogue scale (VAS) pain at 24 weeks than HA, but the between-group difference in Western Ontario and McMaster Universities Osteoarthritis Index (WOMAC) pain score was not statistically significant [29].

In a meta-analysis restricted to Level I and II RCTs, Belk et al. concluded that PRP and HA yield similarly beneficial short-term outcomes [8], and Sambe et al. reached the same conclusion in a 2023 pooled analysis of seven trials [29]. A 2026 double-blind RCT meta-analysis by Wu et al. found a significant WOMAC improvement with PRP relative to controls in primary OA, but only in patients younger than 60 years, illustrating that patient-level moderators may be more important than the intervention itself [30]. The most important caveat comes from Gazendam et al., whose Bayesian network meta-analysis found that saline placebo was statistically indistinguishable from CS, HA, and PRP for pain at up to six months [4]. Because most hip PRP trials used HA rather than saline as a comparator, the possibility that the observed improvements substantially reflect the placebo, needling, and natural history has not been excluded.

### 4.2. PRP Formulation Subgroups in the Hip

The systematic review by Garcia et al. of RCTs on PRP for intra-articular hip disorders addressed preparation methods explicitly, and found that reporting was inconsistent across studies, with variability in leukocyte content, activation, and dosing that precluded confident attribution of outcome differences to any single formulation variable [5]. Within the hip literature, LP-PRP has been used in the more recent intra-articular trials [28], reflecting the general concern that leukocytes may amplify catabolic signalling within a synovial joint, whereas LR-PRP has been used deliberately in the tendinopathy trials described below [31,32], reflecting the opposing view that leukocytes contribute to the reparative response in the tendon. This anatomically split practice is biologically coherent but has not been tested by direct randomized comparison of LR-PRP with LP-PRP at the same hip site.

### 4.3. Greater Trochanteric Pain Syndrome and Gluteal Tendinopathy

The gluteal tendinopathy literature illustrates most clearly how comparator choice determines conclusions. Against active comparators, PRP consistently outperforms corticosteroid. Against a placebo, the benefit disappears. GTPS caused by gluteus medius and minimus tendinopathy is the extra-articular hip condition with the strongest randomized PRP literature. Fitzpatrick et al. randomized 80 patients to a single ultrasound-guided intratendinous LR-PRP injection versus a single CS injection and reported significantly greater improvement in modified Harris Hip Score (mHHS) at 12 weeks in the PRP arm [31]; at two years, the LR-PRP benefit was sustained, whereas the CS effect peaked at six weeks and was not maintained beyond 24 weeks [32]. Jacobson et al. compared percutaneous tendon fenestration with PRP injection and found both to improve symptoms [30]. Atilano et al. compared subfascial PRP injection with enthesis needling in 92 patients and found a greater gain in the Hip Outcome Score Sports Subscale in the PRP arm [33,34].

Against these positive active-comparator trials stands the only adequately powered placebo-controlled evidence: the HIPPO trial (Atcha et al., 79 patients) compared LR-PRP with saline and found no significant difference on the iHOT-12, VAS, mHHS, or EQ-5D at any follow-up point [35]. This is the single most consequential trial in the hip PRP tendinopathy literature. Its result suggests that the apparent superiority of PRP over CS in earlier trials may reflect the known deterioration of CS-treated tendons, rather than representing a true regenerative effect of PRP. A stage-adjusted systematic review of gluteal tendinopathy treatment reached a correspondingly cautious position on the place of injectable biologics [36], and a broader meta-analysis of PRP across tendinopathies found effects that were positive in aggregate, but heterogeneous and formulation-dependent [37].

### 4.4. Femoroacetabular Impingement Syndrome and Hip Arthroscopy

PRP has been used as an intraoperative adjunct during arthroscopic labral repair and femoral neck osteoplasty for FAIS. Foo et al. conducted a prospective randomized single-blinded trial in 113 patients aged 16–50 years; at a minimum of two years, there was no significant between-group difference in change in iHOT-12, Non-Arthritic Hip Score, or Hip Disability and Osteoarthritis Outcome Score—Short Form compared with saline placebo [38]. The systematic review by Garcia et al. likewise found conflicting patient-reported outcomes across trials, and could not identify a preparation protocol associated with benefit [5]. On present evidence, routine peri-operative PRP in hip arthroscopy is not supported. (Table 2).

## 5. Bone Marrow Aspirate Concentrate in Hip Disorders

### 5.1. Intra-Articular BMAC for Hip Osteoarthritis

The intra-articular hip BMAC literature is small and uncontrolled. Rodriguez-Fontan et al. prospectively followed 25 joints (15 hips, 10 knees) treated with intra-articular BMAC for early OA and reported satisfactory results in approximately 63% of patients, with two hips converting to THA within eight months [39]. Whitney et al. prospectively enrolled 24 patients with symptomatic hip OA who elected a single intra-articular bone marrow concentrate injection and reported short-term improvement in pain and function; the design was a case series, Level of Evidence 4 [40]. A systematic review identified only five clinical studies of intra-articular BMAC for hip OA in total, comprising 182 patients across all studies, with no reported adverse events and consistent but uncontrolled reports of reduced pain and improved function [6].

Three features of this literature deserve emphasis. First, there is no randomized, placebo-controlled trial of intra-articular BMAC for hip OA. Second, sample sizes are small and follow-up short, so any inference about disease modification is unsupported. Third, none of these studies reported structural imaging endpoints capable of testing the cartilage-preservation rationale that motivates cell-based therapy in the first place. Given that saline injection alone produces measurable symptomatic improvement in hip OA [4], uncontrolled before-and-after improvement in these cohorts cannot be attributed to the biologic.

### 5.2. Osteonecrosis of the Femoral Head

ONFH is where BMAC in the hip has its strongest biological rationale and its largest amount of clinical literature. Hernigou and Beaujean established the stage-dependence that has governed all subsequent interpretation: outcomes are substantially better in pre-collapse disease (ARCO stages I–IIIa) than in post-collapse hips [7]. Gangji et al. conducted a blinded controlled pilot demonstrating reduced pain and delayed progression with bone marrow implantation compared with CD alone [41], with benefit maintained at five years [42]. Sen et al. randomized patients to CD alone versus CD with instillation of bone marrow mononuclear cells, and reported early advantages for the cell-augmented arm [43], and Tabatabaee et al. reached similar conclusions in a comparative study of early-stage disease [44].

The biological basis for stage-dependence is mechanistically coherent. In pre-collapse disease (ARCO stages I–IIIa), the subchondral architectural integrity is preserved and residual host bone provides a scaffold onto which marrow-delivered progenitors can engraft, migrate into the necrotic zone, and participate in bone repair. In post-collapse disease (ARCO stage III–IV), the subchondral plate is fractured, the articular surface is irreversibly deformed, and the mechanical environment is hostile to cell engraftment; structural restoration cannot be achieved by cell delivery alone. This mechanistic argument contextualises why the Hauzeur et al. double-blind RCT was negative in stage 3 disease [45,46] while earlier controlled studies reported benefit in pre-collapse cohorts. The counterweight is Hauzeur et al., who found no improvement in disease evolution compared with CD alone in stage 3 (post-collapse) ONFH [46]. Pepke et al. performed a randomized prospective study of CD with BMAC versus CD without BMAC in 24 patients, assessing pain, Harris Hip Score, MRI necrosis volume, and in vitro colony-forming unit–fibroblast (CFU-F) counts [45].

Cellular product heterogeneity further limits inference across studies. The marrow-derived products used in published ONFH trials have ranged from unfractionated whole aspirate to density-gradient concentrated BMAC to culture-expanded mesenchymal stromal cells, and nucleated cell counts have rarely been reported. Piuzzi et al. documented the fact that only a minority of published ONFH cell therapy trials provided meaningful product characterisation, making dose–response inference impossible [47,48,49]. Read together with the stage-dependence established since Hernigou’s original series, the reasonable synthesis is that marrow-derived cell augmentation of CD may benefit pre-collapse disease and does not benefit post-collapse disease.

This synthesis is now supported by guideline-level evidence. The 2024 Bayesian network meta-analysis by Wang et al. (Stem Cell Res Ther) found BMAC combined with bone grafting to be among the most favourable strategies for pre-collapse ONFH among six regenerative techniques [50,51,52,53,54,55]. A 2025 systematic review and meta-analysis by Tang et al. encompassing 954 subjects found that regenerative therapy with augmented CD reduced disease progression significantly in pre-collapse disease, with BMAC providing among the larger absolute risk reductions [56]. Most recently, the 2026 Association Research Circulation Osseous (ARCO) international evidence-based clinical practice guideline for ONFH and its commissioned systematic review (Parikh et al., Med Sci 2026 [54]) both concluded that core decompression plus bone marrow concentrate reduces the risk of femoral head collapse compared with core decompression alone (pooled relative risk [RR], 0.55; 95% CI, 0.36–0.83), but rated this evidence as very low certainty, using GRADE methodology—consistent with our overall characterisation. This is consistent with pooled analyses [47,48].

### 5.3. Absence of Standalone Randomized Evidence

It is worth stating plainly that across the entire hip literature, BMAC randomized evidence exists only as an intraoperative adjunct to core decompression in ONFH. There is no randomized trial of BMAC as a standalone intra-articular injection for hip OA, none for gluteal tendinopathy, and none in hip arthroscopy. Any clinical positioning of BMAC for these hip indications is therefore extrapolated—from knee data, from ONFH surgical data, or from mechanisms—rather than demonstrated. Table 3 summarizes the hip BMAC evidence.

## 6. Direct Comparison: What Is and Is Not Known

No published RCT compares PRP with BMAC for any hip disorder. The closest randomized evidence comes from the knee, where Belk et al. systematically reviewed Level I studies and concluded that patients receiving either PRP or BMAC achieved better outcomes than those receiving HA [9]. That analysis did not establish superiority of one biologic over the other, and knee findings cannot be transposed to the hip: the hip is a deeper, more constrained, higher-load joint with different synovial volume, different injection accuracy without imaging guidance, and a different natural history of structural progression.

Comparing Incomparables: Administration Route and Target Pathology as Confounders. A second, more fundamental asymmetry limits any indirect comparison. The PRP hip literature is dominated by intra-articular and peritendinous injection in OA and tendinopathy; the BMAC hip literature is dominated by intralesional bone augmentation in ONFH. These do not deal with the same clinical question, and the products are not being asked to do the same biological work. PRP is being evaluated as an anti-inflammatory and trophic agent in a synovial or tendinous environment; BMAC is being evaluated as an osteoprogenitor delivery vehicle in a necrotic bone compartment. Comparing pooled effect sizes across these two bodies of evidence would be a category error, and any narrative conclusion that one agent is ‘more regenerative’ than the other in the hip is, at present, a mechanistic assertion unsupported by clinical comparative data.

Three consequences follow. First, claims that BMAC is stronger or more regenerative than PRP in the hip are mechanistic assertions, rather than clinical findings. Second, the higher cost, procedural burden, and harvest morbidity of BMAC are not currently justified by comparative hip outcome data. Third, the field needs an adequately powered, standardized, placebo-controlled, three-arm comparison within a single disorder, ideally hip OA or GTPS, where both agents could feasibly be tested against each other and against saline.

## 7. Guidelines, Regulation, and Practical Considerations

### 7.1. Current Guideline Positions

Guideline positions are more restrictive than practice patterns. The 2019 ACR/Arthritis Foundation guideline, based on a systematic review of randomized trials available at that time, strongly advises against PRP for knee and/or hip OA, citing heterogeneity and lack of standardization in available preparations [2]. The strength of this recommendation (strongly against) reflects the guideline panel’s judgment that the evidence available through the review period did not support benefit and that preparation heterogeneity precluded reliable conclusions about any specific product. It is important to note that this guideline pre-dates the HIPPO trial ([35]) and the Okanoue 2025 RCT; however, as discussed above, both of these newer trials are broadly consistent with, rather than contradictory to, the guideline’s position—the HIPPO trial is not against the placebo, and the Okanoue RCT found no statistically significant WOMAC difference. Separately, the 2019 ACR/AF guideline strongly advises against intra-articular HA for hip OA. The 2019 OARSI recommendations centre hip management on arthritis education and structured land-based exercise, with intra-articular CS conditionally recommended for short-term relief and no endorsement of PRP or cell therapy for the hip [3]. For ONFH, the 2026 ARCO international evidence-based clinical practice guideline [53]—the first GRADE-based international guideline for this condition—and its commissioned systematic review [54] acknowledge that core decompression plus bone marrow concentrate is associated with a lower risk of femoral head collapse than core decompression alone (pooled RR 0.55; 95% CI, 0.36–0.83), but rate this evidence as very low certainty, reflecting the small sample sizes and methodological limitations of the included studies. (Table 4) For marrow-derived products, the AAOS technology overview addressed the knee rather than the hip, and concluded from a systematic review of 12 articles that the evidence base remained limited [50].

### 7.2. Regulatory and Practical Issues

Both products are prepared at the point of care from autologous tissue, which places them in a regulatory category distinct from cultured cell therapies but does not exempt them from oversight, and requirements differ substantially between jurisdictions. Clinicians should be explicit with patients that neither agent is guideline-endorsed for hip indications, that both are typically self-funded, and that BMAC carries additional procedural burden. Practical differences matter: PRP requires only venipuncture and a single visit, whereas BMAC requires marrow aspiration under local or regional anaesthesia with attendant harvest-site discomfort. Because the hip joint cannot be reliably entered by landmark palpation, image guidance—ultrasound or fluoroscopy—should be considered mandatory for intra-articular delivery in this joint, and this should be stated in trial reports.

## 8. Limitations

This review is narrative, rather than systematic. No protocol was registered, screening and data extraction were not duplicated, and no formal risk-of-bias or Grading of Recommendations Assessment, Development and Evaluation (GRADE) appraisal was undertaken. The absence of formal risk-of-bias assessment using tools such as the Cochrane RoB 2.0 or ROBINS-I means that internal validity differences between cited studies were not systematically quantified, and selection bias in the studies cited cannot be excluded. Our narrative synthesis may have over-represented positive findings from smaller, uncontrolled studies—particularly in the BMAC hip OA literature, where all available evidence is of Level 4—relative to null findings from larger, blinded trials. The absence of duplicate screening means reviewer disagreements in study selection were not captured. For clinical readers, the certainty of conclusions drawn from this review should be regarded as hypothesis-generating, rather than definitive; these limitations are inherent to the narrative review format and are consistent with similar reviews published in *Bioengineering* and comparable journals. English-language restriction may have omitted relevant work. No quantitative pooling was performed, and the narrative structure means that studies of very different design—double-blind placebo-controlled RCTs and uncontrolled case series—are discussed within the same sections, albeit with their design stated. Most importantly, the underlying literature itself constrains what any review can conclude: PRP and BMAC products were inconsistently characterized across studies, comparators varied (saline, HA, CS, needling, and surgery alone), follow-up was generally short, and structural endpoints were rarely reported. Readers should treat the comparative statements in this review as a map of the evidence landscape, rather than as a treatment recommendation.

## 9. Research Agenda and Recommended Trial Design Standards

The methodological deficiencies identified across Section 4, Section 5 and Section 6 translate directly into specific trial design requirements for the next generation of hip orthobiologic research. Rather than generic recommendations, the following requirements are each motivated by a specific gap in the current evidence. The methodological requirements for the next generation of hip orthobiologic trials follow directly from the deficiencies identified above.

Product characterisation is the most fundamental unmet requirement. For PRP, investigators must report absolute platelet dose per injection (in platelets × 10^9^/mL and total platelets delivered), leukocyte and erythrocyte content (absolute count and proportion of baseline), activation protocol (thrombin, calcium chloride, freeze-thaw, or none), injected volume, number of injections, and the specific commercial system used. For BMAC, investigators must report aspirate volume per withdrawal per site, harvest site (posterior versus anterior iliac crest), nucleated cell count (NCC), colony-forming unit–fibroblast (CFU-F) density or CD34+ cell fraction as a progenitor surrogate, and the specific concentration system. Without these data, neither dose–response relationships nor inter-study comparisons are possible. This requirement is motivated directly by the systematic reviews of Chahla et al. [16], Piuzzi et al. [22], and Murray et al. [23], each of which found product characterisation to be reported in fewer than one-third of published studies.

Trial design should, wherever feasible, adopt a three-arm parallel structure: PRP versus BMAC versus saline placebo, powered at 80% for a pre-specified minimal clinically important difference (MCID) on a validated hip-specific patient-reported outcome measure. This design is motivated by two findings: first, the Gazendam network meta-analysis demonstrated that saline is statistically indistinguishable from active comparators in hip OA [4], meaning that any future two-arm trial using HA or CS as comparator cannot exclude placebo effect; second, no head-to-head PRP versus BMAC trial exists, making the three-arm design the most efficient use of a study population. Mandatory blinding of the participant, the outcome assessor, and—using image-guided injection with identical procedural protocol—the injector, should be preregistered. All hip intra-articular injections must use image guidance (ultrasound or fluoroscopy), as landmarked hip injection is unreliable; the guidance modality and confirmation method must be reported.

Imaging endpoints should be mandatory for any trial claiming disease modification. In hip OA, cartilage morphology by delayed gadolinium-enhanced MRI of cartilage (dGEMRIC) or T2 mapping represents the minimum structural standard; joint-space-width measurement by radiography is insufficient for short-duration trials. In gluteal tendinopathy, tendon structural integrity by ultrasound tissue characterisation (UTC) or MRI provides objective healing confirmation. In ONFH, necrotic volume by MRI and the rate of femoral head collapse are established structural endpoints that must accompany patient-reported outcomes.

Follow-up should be a minimum of 24 months, with conversion to THA or further surgical intervention—including repeat injection—reported as pre-specified hard endpoints. This requirement is motivated by the Fitzpatrick 2019 [32] two-year follow-up data, which showed that the CS advantage reversed relative to PRP by 24 weeks; short follow-up in CS-controlled trials therefore systematically overestimates the difference between PRP and CS at longer time points.

Reporting standards require pre-registration of the protocol and publication of the full protocol paper before enrolment. Adherence to the CONSORT extension for orthobiologics—currently in development—should be stipulated. Subgroup analyses by PRP formulation (LP-PRP versus LR-PRP) and by BMAC progenitor yield must be pre-specified, not post hoc. Future trials should also report patient-level moderators: age (given the Wu 2026 [51] finding that WOMAC benefit was restricted to patients under 60), sex, OA grade, and ARCO stage for ONFH. (Table 5).

## 10. Conclusions

PRP rests on a numerically larger, but methodologically heterogeneous and comparator-sensitive, evidence base for hip disorders. Its apparent advantage over HA in hip OA is not confirmed against saline, and its apparent advantage over corticosteroid in gluteal tendinopathy was not reproduced in the one adequately powered placebo-controlled trial. BMAC is biologically appealing and has its most credible hip role as augmentation of core decompression in pre-collapse osteonecrosis of the femoral head, where benefit is stage-dependent, absent in post-collapse disease, and now acknowledged in the 2026 ARCO international clinical practice guideline with a very low certainty rating; as a standalone intra-articular injection for hip osteoarthritis, it remains clinically under-evidenced, relying on small uncontrolled cohorts. No randomized trial has compared the two agents for any hip disorder, and neither is established as superior. A critical conceptual contribution of this review is the recognition that PRP and BMAC inhabit almost entirely separate hip-specific evidence bases—each addressing a different disorder with a different mode of administration—making direct comparison methodologically impossible with current data. Both remain outside current society recommendations for the hip. Standardized, hip-specific, placebo-controlled head-to-head RCTs—incorporating full biologic characterization, structural imaging endpoints, and follow-up of at least two years—represent the single most important research priority in this field.

## Figures and Tables

**Table 1:** Comparative characteristics of platelet-rich plasma (PRP) and bone marrow aspirate concentrate (BMAC) relevant to hip applications. Abbreviations: HLA-DR, human leukocyte antigen-DR; IL-1Ra, interleukin-1 receptor antagonist; MSC, mesenchymal stromal cell; ONFH, osteonecrosis of the femoral head; PDGF, platelet-derived growth factor; TGF-β, transforming growth factor-β; VEGF, vascular endothelial growth factor.

Characteristic	PRP	BMAC
Source tissue	Peripheral venous blood	Bone marrow, usually posterior superior iliac spine
Principal active components	Platelets and α-granule growth factors (PDGF, TGF-β, VEGF, others); plasma proteins	Nucleated marrow cells, hematopoietic and connective tissue progenitors, platelets, IL-1Ra, broad soluble milieu
MSC content	Absent	Present, but a small minority of nucleated cells; not a purified MSC product
Harvest morbidity	Venipuncture only	Aspiration site pain; requires local or regional anaesthesia; occasional harvest-site complications
Principal sources of variability	Platelet dose, leukocyte and erythrocyte content, activation, volume, injection number, kit	Aspiration volume and technique, harvest site, donor age and comorbidity, concentration device
Classification frameworks available	PAW, PLRA, DEPA, MARSPILL, ISTH scheme	No widely adopted product classification; minimum reporting standards proposed only
Dominant hip application in published studies	Intra-articular injection for hip OA; peritendinous injection for gluteal tendinopathy; intraoperative use in hip arthroscopy	Intralesional augmentation of core decompression for ONFH; small intra-articular series for hip OA
Relative cost and logistics	Lower; single-visit, point-of-care	Higher; procedural suite, greater consumable and time cost

**Table 2:** Principal clinical studies of platelet-rich plasma (PRP) in hip disorders. Certainty column added per reviewer request. Abbreviations: CS, corticosteroid; DDH, developmental dysplasia of the hip; FAIS, femoroacetabular impingement syndrome; GTPS, greater trochanteric pain syndrome; HA, hyaluronic acid; iHOT-12, International Hip Outcome Tool-12; LP-PRP, leukocyte-poor PRP; LR-PRP, leukocyte-rich PRP; mHHS, modified Harris Hip Score; NMA, network meta-analysis; OA, osteoarthritis; RCT, randomized controlled trial; VAS, visual analogue scale; WOMAC, Western Ontario and McMaster Universities Osteoarthritis Index.

Study	Design	Condition	Comparator	Principal Finding	Certainty (GRADE-Informal)
Battaglia 2013 [24]	RCT	Hip OA	HA	Comparable improvement	Very low
Dallari 2016 [25]	RCT (3 arms)	Hip OA	HA; PRP + HA	PRP favourable on pain; combination gave no added benefit	Very low
Di Sante 2016 [26]	RCT	Hip OA	HA	Early pain advantage not sustained	Very low
Doria 2017 [27]	RCT	Early hip OA	HA	Similar improvement in both arms	Very low
Kraeutler 2021 [28]	Double-blind pilot	Hip OA	Low-MW HA	LP-PRP not superior	Low
Okanoue 2025 [29]	Double-blind RCT	Hip OA (DDH)	HA	Greater VAS improvement; WOMAC NS	Low
Belk 2022 [8]	Meta-analysis (Level I–II RCTs)	Hip OA	HA	Similarly beneficial short-term outcomes	Moderate
Gazendam 2021 [4]	Bayesian NMA	Hip OA	Saline, CS, HA	Saline indistinguishable from PRP	Moderate
Garcia 2020 [5]	Systematic review	FAIS, labral, hip OA	Various	Inconsistent preparation reporting; conflicting outcomes	Very low
Fitzpatrick 2018 [31]	Double-blind RCT (*n* = 80)	Gluteal tendinopathy	Single CS	Greater mHHS at 12 weeks with LR-PRP	Moderate
Fitzpatrick 2019 [32]	2-year follow-up of above	Gluteal tendinopathy	Single CS	LR-PRP benefit sustained; CS effect lost	Moderate
Atilano 2024 [33]	RCT (*n* = 92)	GTPS	Enthesis needling	Greater sports-subscale gain with PRP	Low
Atcha 2025 [35]	Double-blind placebo RCT (*n* = 79)	Refractory GTPS	Saline placebo	No significant difference; routine use not supported	Moderate
Foo 2021 [38]	Randomized (*n* = 113)	FAIS, hip arthroscopy	Saline placebo	No between-group difference at min 2 years	Low

**Table 3:** Principal clinical studies of bone marrow aspirate concentrate (BMAC) and related marrow-derived cell products in hip disorders. Certainty column added per reviewer request. Abbreviations: ARCO, Association Research Circulation Osseous; BMAC, bone marrow aspirate concentrate; CD, core decompression; MRI, magnetic resonance imaging; NMA, network meta-analysis; OA, osteoarthritis; ONFH, osteonecrosis of the femoral head; RCT, randomized controlled trial; THA, total hip arthroplasty.

Study	Design	Condition	Comparator	Principal Finding	Certainty (GRADE-Informal)
Hernigou 2002 [7]	Large clinical series	ONFH	None	Outcome stage-dependent; established technique	Very low
Gangji 2004 [41]	Blinded controlled pilot	Early ONFH	CD alone	Reduced pain and delayed progression	Low
Gangji 2011 [42]	5-year prospective controlled	Early ONFH	CD alone	Benefit maintained at 5 years	Low
Sen 2012 [43]	RCT	ONFH	CD alone	Early advantage for cell instillation	Low
Tabatabaee 2015 [44]	Comparative study	Early-stage ONFH	CD alone	Improved outcome	Very low
Pepke 2016 [45]	Randomized prospective (*n* = 24)	Non-traumatic ONFH	CD alone	Safety confirmed; clinical and MRI outcomes assessed	Low
Hauzeur 2018 [46]	Double-blind RCT	Stage 3 ONFH	CD alone	No improvement in disease evolution	Moderate
Wu 2026 [51]	Double-blind RCT meta-analysis	Primary and post-traumatic OA	Controls	WOMAC improvement significant in patients <60 years only	Moderate
Wang 2024 [55]	Bayesian NMA (Stem Cell Res Ther)	Pre-collapse ONFH	6 regenerative techniques vs. CD	BMAC + bone graft among most favourable strategies	Very low
Tang 2025 [56]	Systematic review and MA (*n* = 954)	Pre-collapse ONFH	CD alone	Regenerative augment reduces disease progression	Low
ARCO 2026 [53,54]	International CPG/systematic review	ARCO stages I–III ONFH	CD alone	CD + BMC reduces collapse risk (RR 0.55; 95% CI 0.36–0.83); very low certainty	Very low
Jindal 2021 [47]	Systematic review and MA	Post-collapse ONFH	CD alone	Limited benefit post-collapse	Low
Rodriguez-Fontan 2018 [39]	Prospective cohort (15 hips)	Early hip OA	None	Satisfactory in ~63%; two THA by 8 months	Very low
Whitney 2020 [40]	Prospective case series (*n* = 24)	Symptomatic hip OA	None	Short-term improvement in pain and function	Very low
Chandrashekar 2024 [6]	Systematic review	Hip OA	None	5 studies, 182 patients; no RCT; no adverse events	Very low

**Table 4:** Society guidance relevant to platelet-rich plasma (PRP) and bone marrow aspirate concentrate (BMAC) in the hip. ARCO 2026 row added per reviewer request. Abbreviations: AAOS, American Academy of Orthopaedic Surgeons; ACR, American College of Rheumatology; ARCO, Association Research Circulation Osseous; BMC, bone marrow concentrate; CD, core decompression; GRADE, Grading of Recommendations Assessment, Development and Evaluation; HA, hyaluronic acid; OA, osteoarthritis; OARSI, Osteoarthritis Research Society International; RR, relative risk.

Source	Scope	Position Relevant to Hip Biologics
ACR/Arthritis Foundation 2019 [2]	Hand, hip, and knee OA	PRP strongly advised against for knee and/or hip OA; intra-articular HA strongly advised against for hip OA; no recommendation on BMAC. Review pre-dates 2025 trial data; newer evidence is broadly consistent.
OARSI 2019 [3]	Knee, hip, and polyarticular OA	Core treatment is education plus structured land-based exercise; intra-articular CS conditionally recommended for short-term relief; PRP and cell therapy not endorsed for hip.
AAOS technology overview [50]	Concentrated bone marrow aspirate, knee OA	Evidence base judged as limited; no hip-specific recommendation exists.
ARCO International CPG 2026 [53,54]	Non-traumatic ONFH, ARCO stages I–III	CD + bone marrow concentrate associated with lower collapse risk than CD alone (pooled RR 0.55; 95% CI 0.36–0.83); evidence rated very low certainty by GRADE. No recommendation on standalone intra-articular BMAC for hip OA.

**Table 5:** Summary of minimum requirements for future hip orthobiologic trials. Substantially expanded per reviewer request. Abbreviations: ARCO, Association Research Circulation Osseous; BMAC, bone marrow aspirate concentrate; CFU-F, colony-forming unit–fibroblast; CONSORT, Consolidated Standards of Reporting Trials; CS, corticosteroid; dGEMRIC, delayed gadolinium-enhanced MRI of cartilage; MCID, minimal clinically important difference; MRI, magnetic resonance imaging; ONFH, osteonecrosis of the femoral head; PRP, platelet-rich plasma; RCT, randomized controlled trial; THA, total hip arthroplasty; UTC, ultrasound tissue characterisation.

Domain	Minimum Requirement	Motivation from Current Evidence
Product characterisation—PRP	Absolute platelet dose, leukocyte and erythrocyte content, activation protocol, injected volume, number of injections, commercial system	Chahla et al. 2017 [16]: fewer than one-third of studies reported full composition
Product characterisation—BMAC	Aspirate volume per site, harvest site, NCC, CFU-F or CD34+ fraction, concentration system	Piuzzi et al. 2018 [22]; Murray et al. 2018 [23]: no study reported detail sufficient for replication
Trial design	Three-arm RCT (PRP vs. BMAC vs. saline placebo); 80% power for pre-specified MCID; blinding of participant, assessor, injector	Gazendam 2021 [4]: saline indistinguishable from active comparators; no head-to-head PRP vs. BMAC trial exists
Image guidance	Mandatory ultrasound or fluoroscopy for all intra-articular hip injections; modality and confirmation method reported	Hip joint not reliably entered by landmark palpation
Imaging endpoints	dGEMRIC or T2 mapping for OA; UTC or MRI for tendinopathy; MRI necrotic volume and collapse rate for ONFH	No current hip trial has tested structural modification
Follow-up	Minimum 24 months; THA or re-injection as pre-specified hard endpoints	Fitzpatrick 2019 [32]: CS benefit reversed relative to PRP by 24 weeks; short follow-up distorts comparisons
Reporting	Protocol pre-registration; CONSORT extension for orthobiologics; pre-specified subgroups by formulation and progenitor yield	Systematic under-reporting identified across all cited reviews
Patient-level moderators	Age (<60 vs. ≥60 years), sex, OA grade, ARCO stage	Wu 2026 [51]: WOMAC benefit restricted to patients under 60; stage dependence in ONFH [7,46]

## Data Availability

No new data were created or analysed in this study. Data sharing is not applicable to this article.
